# Innate and adaptive immune responses to dead and dying cells

**DOI:** 10.1186/ar3587

**Published:** 2012-02-09

**Authors:** Keith B Elkon, Yueng Peng

**Affiliations:** 1 Division of Rheumatology, University of Washington, Seattle, WA 98195, USA; 2 Department of Immunology, University of Washington, Seattle, WA 98195, USA

## 

Under steady state conditions, billions of dead and dying cells are removed by extrusion from epithelial surfaces as well as by phagocytosis. Cells such as macrophages and dendritic cells have specialized receptors that directly recognize altered protein or lipids on apoptotic cells or opsonins that bind to the dying cell. Once engulfed, phagosomes containing apoptotic cells are rapidly acidified and the contents degraded by proteases and nucleases in lysozymes. During necrosis, cellular material is released prior to engulfment and extracellular nucleases as well as intracellular sensors dictate the inflammatory potential of the cellular debris. The outcome may be release of TNF-a, IL-1-b or interferon (IFN)-a depending upon the type of phagocyte, molecular nature of the cellular particle and the intracellular sensor engaged.

In addition to responses by cells of the innate immune system, we have recently defined a link between processing of apoptotic cells and their debris to T cell activation [1]. MFG-E8 is an opsonin (bridging protein) that binds to phosphatidylserine on apoptotic cells and facilitates their removal through interaction with integrins on phagocytes. Mice deficient in MFG-E8 develop lupus like autoimmunity associated with accumulation of apoptotic cells in vivo. We observed that older MFG-8-/- mice spontaneously developed a dermatitis associated with CD8 T cell infiltration and striking activation of effector memory CD8 T cells. T cell responses to both exogenous and endogenous apoptotic cell associated antigens were enhanced in MFG-E8 deficient mice and transfer of ovalbumin (OVA) reactive OT-I CD8 T cells caused accelerated diabetes in MFG-E8-/- RIP-mOVA mice and skin disease in kmOVA transgenic mice. The enhanced CD8 T cell response was attributed to increased cross-presentation by dendritic cells (DCs) associated with increased detection of antigen peptide MHCI complexes. Investigation of intracellular trafficking revealed that, whereas intact apoptotic cells ingested by wild type DC rapidly fused with lysosomes, in the absence of MFG-E8, smaller apoptotic cell fragments persisted in endosomal compartments and failed to fuse with lysosomes.

These observations suggest that in addition to altering the rate of clearance of apoptotic cells, MFG-E8 deficiency promotes immune responses to self antigens by altered intracellular processing leading to enhanced antigen presentation. Thus, handling of dead and dying cells impacts both innate and adaptive immune responses to self antigens.
